# Empowering the Vulnerable: The Impact of SEL on Traumatized Children’s Academic and Social Outcomes in Crises

**DOI:** 10.1007/s11920-024-01555-8

**Published:** 2024-11-11

**Authors:** Anies Al-Hroub, Rawand Al-Hroub

**Affiliations:** 1 Department of Education, American University of Beirut, Beirut, Lebanon; 2 Psychology Department, Royal Holloway University of London, Egham, UK

**Keywords:** Social and Emotional Learning (SEL). Trauma. Academic Outcomes. Social Integration. Resilience. Cultural Adaptation. Crisis Intervention. Parental Involvement

## Abstract

**Purpose of Review:**

This article critically reviews the impact of Social and Emotional Learning (SEL) on traumatized children’s academic and social outcomes, focusing on studies from the past three years across diverse contexts. It emphasizes the need for culturally sensitive, trauma-informed programs that cater to various settings, while also exploring the critical roles of parents and educators in SEL implementation. Additionally, the paper highlights challenges in deploying SEL in crisis-affected regions and offers strategies to address systemic barriers.

**Recent Findings:**

Recent studies show that SEL interventions significantly foster emotional regulation, resilience, empathy, and social integration, vital for personal well-being and successful social adaptation. However, the effect of SEL on academic performance, particularly in literacy and numeracy, remains inconsistent. Cultural adaptation of SEL programs is critical to their success, especially in non-Western settings, with a growing focus on trauma-informed approaches to address the needs of children affected by crises, including the COVID-19 pandemic. Despite these benefits, challenges such as resource limitations, lack of policy support, and the need for educator training continue to impede effective SEL implementation.

**Summary:**

SEL offers a vital framework for supporting traumatized children, particularly in crisis-affected regions. This review explores five key themes: the enhancement of emotional regulation and social integration, the necessity of cultural adaptation, the involvement of parents and educators, the challenges of implementing SEL in crisis contexts, and the importance of developing trauma-informed policies. The article also identifies future research directions, emphasizing the need for systemic reforms and culturally adapted SEL models.

## Introduction

The integration of Social and Emotional Learning (SEL) into the fabric of educational and therapeutic interventions represents a paradigm shift in addressing the holistic needs of children, particularly those who have experienced trauma. In the wake of escalating global conflicts, migration crises, and the pervasive impacts of pandemics, the urgency for effective, comprehensive interventions has never been more acute. Traumatized children, often bearing the invisible scars of their experiences, present unique challenges that extend beyond the conventional educational frameworks, necessitating a multi-dimensional approach to their rehabilitation and growth [1, 2].

SEL offers a promising avenue, emphasizing the cultivation of critical skills such as emotional regulation, empathy, resilience, and social engagement. These competencies are pivotal not only for personal well-being but also for academic success and social integration, providing a foundation for children to navigate their trauma and rebuild their lives [3]. This critical review endeavors to dissect the application of SEL programs for traumatized children, weaving together insights from recent studies to illuminate the efficacy, challenges, and essential considerations for deploying SEL across diverse, often challenging, environments [2].

In this critical review, we delve into the crucial and pressing issue of integrating Social-Emotional Learning (SEL) approaches within the context of child mass trauma, focusing on recent studies published in the past five years. The overarching aim of this paper is to synthesize key findings, identify themes, and evaluate the efficacy and challenges of SEL interventions across diverse settings. This paper aims to: (a) analyze the impact of SEL on the academic performance and social integration of traumatized children, (b) evaluate the importance of cultural sensitivity and the adaptation of SEL programs to the unique needs of children in various contexts, (c) explore the SEL in response to global crises, (d) explore the role of parents and educators in the effective delivery of SEL and its impact on children’s outcomes, (e) discuss the challenges inherent in implementing SEL with vulnerable populations in crisis-affected regions and the strategies to overcome them, and (f) highlight the future directions for research and practice in integrating SEL within educational systems for traumatized children. This analysis is vital for educators, policymakers, and mental health professionals striving to support the well-being and educational outcomes of children who have experienced trauma due to conflict, displacement, or global health crises.

## Critical Review

The studies presented offer a multifaceted view of the challenges and strategies involved in implementing Social-Emotional Learning (SEL) approaches within the context of child mass trauma. These range from refugee crises across various regions to the global impact of the COVID-19 pandemic. The research collectively underscores the complexity of addressing SEL needs in crises, addressing the Impact of SEL on academic and social integration, cultural sensitivity and adaptation of SEL programs, SEL in response to global crises, the role of parents and educators in SEL, and challenges, and complex dynamics in implementing SEL. Thus, we can discern patterns, successes, and gaps in the current SEL efforts through these themes.

### The Impact of SEL on Academic and Social Integration

The application of SEL in educational settings has shown considerable benefits in fostering academic success and promoting social integration, particularly for traumatized children. Dolan’s study [4] on Syrian refugee children in Lebanon demonstrated that integrating SEL with nonformal remedial education significantly improved children’s perceptions of public schools, creating a more inclusive environment that enhanced their sense of belonging and adaptation. However, while SEL improved behavioral regulation and some basic skills, it showed limited impact on broader academic outcomes such as literacy and numeracy. Similarly, Durlak et al. [5] found that SEL programs consistently enhance social-emotional skills, attitudes, and classroom behavior, contributing to overall student well-being and academic performance, which is essential for integrating traumatized students into formal educational settings. Cipriano et al.’s [6] meta-analysis further confirmed that Universal School-Based (USB) SEL interventions significantly improved social-emotional skills, behaviors, and peer relationships. However, their impact on core academic skills remained variable, indicating that SEL’s primary strength is in promoting social adaptation rather than directly boosting academic achievement.

Wigelsworth et al. [7] identified substantial variability in the quality and rigor of SEL research, complicating the ability to draw definitive conclusions. Their review highlighted a lack of clear definitions for “whole-school approaches” versus class-based curricula, making it difficult to evaluate their relative effectiveness. While whole-school SEL interventions are theorized to be more impactful, the evidence remains inconclusive due to inconsistent implementation frameworks and methodological complexities. This calls for a more structured approach in future SEL research to better understand what works and for whom, ensuring that SEL programs are both evidence-based and contextually adaptable.

### Cultural Sensitivity and Adaptation of SEL Programs

The success of SEL programs is intricately tied to their ability to resonate with the cultural and contextual realities of the children they aim to support. Matsuba et al. [8] in Northern Uganda emphasized the necessity of tailoring SEL content to align with the cultural backdrop of the participants, enhancing both the relevance and effectiveness of the interventions. This cultural sensitivity not only fosters a deeper engagement with the SEL material but also affirms the identities and experiences of the children, providing a more empathetic and supportive learning environment.

The research by Ștefan et al. [9] and Fernández-Leyva et al. [10] highlights that the success of SEL interventions is strongly influenced by the cultural context and educational systems in which they are implemented. Ștefan et al.’s [9] systematic review revealed that while some SEL programs show positive outcomes in early childhood settings, only 10.5% met the criteria to be classified as well-established, indicating limited adaptability to diverse cultural and contextual settings. Similarly, Fernández-Leyva et al. examined the impact of nationality on the development of social skills and academic success among immigrant students in Spain, finding that cultural background significantly affects SEL effectiveness, with European-origin students achieving better social and academic outcomes than their Asian and African peers. Similarly, Aziz et al. [11] developed an integrated SEL framework specifically designed for Lebanese schools. Their approach included an extensive review of existing SEL practices, complemented by qualitative insights from educators within the Lebanese education system. The key finding emphasized the necessity of culturally sensitive SEL programs that resonate with the Lebanese educational ethos, suggesting that such tailored programs can significantly enhance students’ social and emotional competencies.

Wigelsworth et al.’s study [7] highlighted an overgeneralization of SEL effectiveness, with limited analysis of its impact on different student subgroups. There was a significant lack of data on how SEL programs influence students based on factors such as socioeconomic status, ethnicity, or initial social-emotional competence. These findings point to the need for culturally tailored SEL programs that cater to the unique needs of diverse student populations to support their optimal social and academic development.

### SEL in Response to Global Crises

The advent of global crises such as the COVID-19 pandemic has compounded the challenges faced by traumatized children, amplifying existing traumas and introducing new ones. Scott et al. [12] exploration of trauma-informed SEL strategies in the wake of the pandemic underscores the need for comprehensive, empathetic approaches that address the multifaceted effects of such crises on child development. Their research highlights SEL’s potential as a vital component of emergency education responses, offering resilience-building strategies that are crucial in navigating the aftermath of global upheavals. Likewise, Durlak et al. [5] reviewed the effectiveness of SEL programming in helping students cope with stressors, such as those resulting from global crises like the COVID-19 pandemic. They found that SEL programs could build resilience and emotional regulation, supporting children through collective traumas.

### The Role of Parents and Educators in SEL

The success of SEL interventions relies heavily on the active involvement of parents and educators, who play pivotal roles in its effective delivery. Görgüner’s [13] examination of the relationship between parental involvement and children’s SEL outcomes in Jordan opens new avenues for understanding the dynamics of SEL in the Syrian refugee context. The absence of a significant relationship in this study contradicts the broader educational literature, which frequently underscores the crucial role of parental engagement. This discrepancy points to the unique challenges of fostering parental involvement in SEL within crisis settings, suggesting that conventional strategies may not be as effective in these contexts. The findings underline the importance of developing innovative approaches to engage parents and communities in SEL, potentially involving more tailored, culturally congruent engagement strategies considering the complexities of refugees and crises. Similarly, Cho et al.‘s [14] insights into teachers’ perspectives on refugee English language learners in early elementary grades emphasize the critical need to equip educators with the training and resources necessary for effective SEL implementation.

### Challenges in Implementing SEL with Vulnerable Populations

Implementing SEL interventions in crisis-affected areas involves navigating multiple socio-educational, cultural, systemic, or structural challenges. For example, Daley and McCarthy’s systematic review study [15] identified significant challenges in implementing SEL programs for students with disabilities, particularly in general educational settings, and resource-limited environments. The review analyzed 166 studies to determine how frequently students with disabilities are included, the degree to which their needs are considered in intervention designs, and whether these interventions are effective for this population. Despite the growing emphasis on SEL in general education, only 19 studies explicitly mentioned including students with disabilities, and even fewer analyzed subgroup effects, highlighting a lack of attention to this vulnerable population.

The study found significant variability in the quality and rigor of SEL research, with ambiguous evidence on the effectiveness of whole-school approaches compared to class-based programs. It highlighted a lack of clarity on defining and implementing whole-school SEL strategies and identified gaps in understanding how SEL impacts diverse student subgroups.

Dalrymple’s [16] study of SEL in East Africa uncovered the complex interplay of power and culture, highlighting tensions between Western-developed SEL frameworks and the lived experiences of refugees in non-Western contexts. This analysis calls for a more nuanced approach to SEL—one that is sensitive to the power dynamics and cultural differences influencing educational interventions in crisis settings.

The work of Scott et al. [12] and Deitz et al. [17] brings to the fore the systemic and structural challenges that impact the efficacy of SEL interventions. The review by Scott et al. [12] of the COVID-19 pandemic’s effects on SEL and ACE concerns underscores the exacerbated vulnerabilities of socially disadvantaged students, highlighting the critical need for trauma-informed educational strategies. Deitz et al.‘s systematic review [17] further illuminates the variable impacts of SEL programs globally and across different settings. This study meticulously synthesized data from various SEL programs, employing rigorous meta-analytic methods to evaluate their impact. More specifically, the findings underscored the positive effects of SEL on academic performance, emotional well-being, and social behavior, reinforcing the argument for integrating SEL into educational curricula universally, particularly to meet marginalized populations’ needs.

These findings suggest that overcoming systemic barriers requires a multifaceted approach that includes policy reform, teacher training, and developing contextually adapted SEL content.

### Directions for Future Research

The future of Social and Emotional Learning (SEL) for children who have experienced trauma requires targeted and nuanced exploration in several key areas. One critical direction is the development and testing of SEL programs that are culturally adapted for children from diverse backgrounds and trauma contexts. Future research needs to investigate how culturally sensitive SEL interventions can be tailored to better meet the emotional and social needs of traumatized children in various regions, particularly in non-Western settings. Such efforts may involve the co-design of SEL frameworks in collaboration with local educators and communities to ensure that these programs are relevant, effective, and contextually appropriate.

There is also a clear need for longitudinal studies that examine the long-term impact of SEL interventions on both academic and social-emotional outcomes for children who have experienced trauma. These studies would help clarify the sustained benefits of SEL over time, particularly in fostering emotional resilience, social integration, and educational success. Understanding these long-term effects is crucial for refining SEL programs and ensuring that they provide lasting support for traumatized children.

Furthermore, research should focus on how trauma-informed SEL practices can be integrated into mainstream educational systems, particularly in areas affected by crises. Investigating the specific mechanisms through which trauma-informed SEL approaches build resilience and help children cope with post-traumatic stress, displacement, or other crises, such as pandemics, is vital. By exploring these mechanisms, researchers can better understand how to optimize SEL programs for children in trauma-affected contexts.

The role of parents and educators in the effective delivery of SEL also warrants further exploration. Future studies should investigate innovative strategies to engage parents, especially in crisis contexts where traditional forms of parental involvement may be less feasible. Additionally, research should assess the impact of specialized SEL training for educators, particularly in refugee and trauma-affected populations, to determine how this training can improve student outcomes and foster a more supportive learning environment.

Finally, there is a need to examine how policy reforms and systemic changes can support the large-scale implementation of SEL programs in schools serving trauma-affected populations. Future research could focus on identifying the most effective policy frameworks and systemic supports that facilitate the integration of SEL into national education curricula, especially in regions impacted by conflict or displacement. Understanding the role of policy and systemic reform is essential for ensuring the widespread and sustainable adoption of SEL programs in schools that serve children dealing with trauma.

Together, these research directions will contribute to a more comprehensive understanding of how SEL can be effectively tailored, implemented, and sustained to support the unique needs of traumatized children across diverse contexts.

## Data Availability

No datasets were generated or analysed during the current study.

## References

[1] Al-Hroub A. Art therapy interventions for Syrian child and adolescent refugees: enhancing mental well-being and resilience. Curr Psychiat Rep. 2003;25:857–63. 10.1007/s11920-023-01474-0. 10.1007/s11920-023-01474-0 37943430

[2] Annous N, Al-Hroub A, El Zein F. A systematic review of empirical evidence on art therapy with traumatized refugee children and youth (2010–2020). Front Psychol. 2022;13:1–10. 10.3389/fpsyg.2022.811515. 10.3389/fpsyg.2022.811515 PMC9189733 35707659

[3] Al-Hroub A, Reis S, Madaus J, Shuayb I. (2023). Serving vulnerable and marginalized populations in social and educational contexts: Challenges and opportunities. Front Psychol. 2023;14: 1310260. 10.3389/fpsyg.2023.1310260 10.3389/fpsyg.2023.1310260 PMC10642171 37965651

[4] •• Dolan CHK. Supporting Syrian refugee children’s academic and social-emotional learning in national education systems: a cluster randomized controlled trial of nonformal remedial support and mindfulness programs in Lebanon. Am Educ Res J. 2022;59:419–60. 10.3102/00028312211062911.

[5] Durlak JA, Mahoney JL, Boyle AE. (2022). What we know, and what we need to find out about universal, school-based social and emotional learning programs for children and adolescents: A review of meta-analyses and directions for future research. Psychol Bull. 2022;148:765–782. 10.1037/bul0000383

[6] Cipriano C, Strambler MJ, Naples LH, Ha C, Kirk M, Wood M, et al. The state of evidence for social and emotional learning: a contemporary meta-analysis of universal school‐based SEL interventions. Child Dev. 2023;94:1181–204. 10.1111/cdev.13968. 37448158 10.1111/cdev.13968

[7] Matsuba MK, Schonert-Reichl KA, McElroy T, Katahoire A. Effectiveness of a SEL/mindfulness program on Northern Ugandan children. Int J Sch Educ Psychol. 2021;9:113–31. 10.1080/21683603.2020.1760977.

[8] Ștefan CA, Dănilă I, Cristescu D. Classroom-wide school interventions for preschoolers’ social-emotional learning: a systematic review of evidence-based programs. Educ Psychol Rev. 2022;34:2971–3010. 10.1007/s10648-022-09680-7.

[9] Fernández-Leyva C, Tomé-Fernández M, Ortiz-Marcos JM. Nationality as an influential variable with regard to the social skills and academic success of immigrant students. Educ Sci. 2021;11:605. 10.3390/educsci11100605.

[10] Aziz C, Harb N, El Ahmadieh S, Kazan W, El Feghaly Y. An integrated social and emotional learning framework for Lebanese schools. In: Cefai C, Regester D, Dirani LA, editors. Social and emotional learning in the Mediterranean: Cross cultural perspectives and approaches. Leiden: Brill; 2020. pp. 83–98. 10.1163/9789004444515_008.

[11] Scott J, Jaber LS, Rinaldi CM. Trauma-informed school strategies for SEL and ACE concerns during COVID-19. Educ Sci. 2021;11:796. 10.3390/educsci1112079.

[12] Gorguner FS. Parental involvement and child social emotional learning among Syrian refugee families in Jordan: Findings from the FIERCE project [Master Thesis]. [Padua]: University of Padova; 2023. 85p. https://thesis.unipd.it/retrieve/91a2bbfc-f4e9-4e77-a6d7-6347115c55ef/SERIN_GORGUNER_FATMA_SELCEN.pdf

[13] Cho H, Wang C, Christ T. Social-emotional learning of Refugee English language learners in early elementary grades: teachers’ perspectives. J Res Child Educ. 2019;33:40–55. 10.1080/02568543.2018.1531449.

[14] Daley SG, McCarthy MF. Students with disabilities in social and emotional learning interventions: a systematic review. Remedial Spec Educ. 2021;42:384–97. 10.1177/0741932520964917.

[15] Wigelsworth M, Verity L, Mason C, Qualter P, Humphrey N. Social and emotional learning in primary schools: a review of the current state of evidence. Br J Educ Psychol. 2022;92:898–924. 10.1111/bjep.12480. 34921555 10.1111/bjep.12480 PMC9540263

[16] Dalrymple K. Critically examining social emotional learning with refugees in East Africa: Tensions, challenges, and complex dynamics. *Current Issues in Comparative Education (CICE)*, 2023;*25*:7–29. Retrieved from: https://files.eric.ed.gov/fulltext/EJ1417467.pdf

[17] Deitz R, Lahmann H, Thompson T, Youth Division. Social and emotional learning (SEL) systematic review. Washington, DC: Office of Sustainable Development, Education and ; 2021 Aug. 136p. https://dexisonline.com/wp-content/uploads/2023/05/Attachment-1-Systematic-Review-Report.pdf

